# The Long Non-Coding RNA SAMMSON Is a Regulator of Chemosensitivity and Metabolic Orientation in MCF-7 Doxorubicin-Resistant Breast Cancer Cells

**DOI:** 10.3390/biology10111156

**Published:** 2021-11-09

**Authors:** Charlotte Orre, Xavier Dieu, Jordan Guillon, Naïg Gueguen, Seyedeh Tayebeh Ahmadpour, Jean-François Dumas, Salim Khiati, Pascal Reynier, Guy Lenaers, Olivier Coqueret, Arnaud Chevrollier, Delphine Mirebeau-Prunier, Valérie Desquiret-Dumas

**Affiliations:** 1 Mitolab Team, Inserm U1083, CNRS 6015, Mito Vasc Institute, SFR ICAT, Angers University, F-49000 Angers, France; charlotte.orre@etud.univ-angers.fr (C.O.); Xavier.Dieu@chu-angers.fr (X.D.); nagueguen@chu-angers.fr (N.G.); salim.khiati@univ-angers.fr (S.K.); pareynier@chu-angers.fr (P.R.); guy.lenaers@inserm.fr (G.L.); arnaud.chevrollier@univ-angers.fr (A.C.); DePrunier@chu-angers.fr (D.M.-P.); 2 Service de Biochimie et Biologie Moléculaire, CHU Angers, F-49933 Angers, France; 3 Paul Papin ICO Cancer Center, CRCINA, INSERM, Angers University, F-49000 Angers, France; jordan.guillon.chum@ssss.gouv.qc.ca (J.G.); olivier.coqueret@univ-angers.fr (O.C.); 4 Nutrition, Croissance et Cancer, Inserm UMR1069, Université de Tours, F-37032 Tours, France; seyedehtayebeh.ahmadpour@etu.univ-tours.fr (S.T.A.); jean-francois.dumas@univ-tours.fr (J.-F.D.); 5 Service de Neurologie, CHU d’Angers, F-49933 Angers, France

**Keywords:** breast cancer, mitochondria, metabolism, complex I, long non-coding RNA, SAMMSON, reactive oxygen species

## Abstract

**Simple Summary:**

Breast cancer is the most common cancer in women, representing about one third of cancers in developed countries. Despite recent advances in diagnostic methods and increasingly early detection, breast cancer recurrence occurs in more than 20% of patients. Chemoresistance represents an important cause of this recurrence, but the mechanisms involved in this phenomenon, are still largely unknown. One feature of chemoresistant cancer cells is the reorientation of the energetic metabolism to sustain cell proliferation. Recently, long non-coding RNAs (lncRNAs) have emerged as important regulators of cellular metabolic orientation. In the present work, we gave special attention to the long non-coding RNA SAMMSON and addressed the role of this lncRNA in metabolic orientation and chemoresistance of doxorubicin-resistant breast cancer cells. The results shed light on the possible modulation of the SAMMSON expression as an innovative therapeutic approach to target chemoresistant cancer cells specifically.

**Abstract:**

Despite improvements in therapeutic strategies for treating breast cancers, tumor relapse and chemoresistance remain major issues in patient outcomes. Indeed, cancer cells display a metabolic plasticity allowing a quick adaptation to the tumoral microenvironment and to cellular stresses induced by chemotherapy. Recently, long non-coding RNA molecules (lncRNAs) have emerged as important regulators of cellular metabolic orientation. In the present study, we addressed the role of the long non-coding RNA molecule (lncRNA) SAMMSON on the metabolic reprogramming and chemoresistance of MCF-7 breast cancer cells resistant to doxorubicin (MCF-7dox). Our results showed an overexpression of SAMMSON in MCF-7dox compared to doxorubicin-sensitive cells (MCF-7). Silencing of SAMMSON expression by siRNA in MCF-7dox cells resulted in a metabolic rewiring with improvement of oxidative metabolism, decreased mitochondrial ROS production, increased mitochondrial replication, transcription and translation and an attenuation of chemoresistance. These results highlight the role of SAMMSON in the metabolic adaptations leading to the development of chemoresistance in breast cancer cells. Thus, targeting SAMMSON expression levels represents a promising therapeutic route to circumvent doxorubicin resistance in breast cancers.

## 1. Introduction

The heterogeneity and adaptative capabilities of tumors that lead to resistance to chemotherapeutic agents remain important issues in cancer treatment [1]. In spite of a large number of identified mechanisms that underlie resistance to anticancer drugs, none of them fully explains the multidrug resistance observed in patients with advanced cancers [2]. Among the chemoresistance mechanisms identified, the composition of the plasma membrane, overrepresentation of efflux pumps, modification of drug metabolism by detoxifying enzymes, increased cell ability to repair DNA damage or tolerate stress conditions and, finally, defects in apoptotic pathways [3] are those that have received the most attention in research to date. More recently, metabolic plasticity, being the ability of cells to switch their metabolism and nutrient use to cope with the specific requirements of cancer cells, has been identified as a crucial parameter governing chemoresistance, but the pathways still need to be elucidated further [4,5].

The most common feature of this metabolic adaptation is a modification of glucose catabolism represented by a switch from oxidative metabolism to aerobic glycolysis, the well-known “Warburg effect” [6]. During this process, the conversion of glucose to lactate implies an increase in glucose uptake to compensate the lower energy production efficiency of the glycolytic metabolism. Tumor cells also use glutamine to promote cell proliferation by supplying glutamate and α-ketoglutarate to the tricarboxylic acid (TCA) cycle [7]. Lipid metabolism is also deeply modified in cancer cells with an upregulation of de novo lipid synthesis to fulfil the requirement of lipid derivatives for membrane synthesis. All these metabolic changes converge in an increase in catabolic pathways to provide intermediate metabolites important for biomass increase. Being at the crossroads of many metabolic pathways, mitochondria act as the main regulator of cellular metabolic fate and play important roles in the metabolic adaptations observed in cancer cells [8].

Cancer resistance to chemotherapies and metabolic reprogramming depends on epigenetic modifications of the chromatin, which participates in all stages of cancer progression [9]. Long non-coding RNAs (lncRNAs), which are functional RNA molecules larger than 200 nucleotides, emerge as important transcriptional and epigenetic regulators of many cellular processes, including cell proliferation, cell fate decision and apoptosis [10]. Due to their important role in cellular homeostasis, aberrant expressions of lncRNAs are now proposed as modulators of physiological cell proliferation, but also of tumor progression, metastasis formation [11,12] and mitochondrial disturbances found in cancer cells. However, no clear link has yet been established between these processes and lncRNA activity.

The SAMMSON (Survival Associated Mitochondrial Melanoma-Specific Oncogenic Non-Coding RNA) lncRNA is overexpressed in various cancer models (melanoma cells [13], oral squamous cell carcinoma [14], hepatocarcinoma [15], gastric cancer [16] and papillary thyroid carcinoma [17]). In melanoma cells, it has been described as an important regulator of cancer cell metabolic flexibility since it has been shown to be involved in the translational coordination between the nuclear and mitochondrial genome [18]. This lncRNA interferes with both cytosolic and mitochondrial translation, on one hand by interacting with the CARF (Collaborator of ARF)/XRN2 (5′-3′Exoribonuclease 2) complex and on the other hand by targeting the protein p32 (C1QBP) [18], a master regulator of mitochondrial oxidative metabolism [19]. In this study, we hypothesize that, due to its important role in metabolic orientation, SAMMSON could be a key player in cancer progression and chemotherapies resistance, two situations exploiting the metabolic vulnerability of the cells.

To explore this point, we evaluated the link between SAMMSON expression, metabolic orientation and chemoresistance in a cellular model of breast cancer, which is the foremost cause of cancer mortality in women worldwide [20], with recurrent incidences occurring in more than 20% of patients [21]. We showed that silencing the SAMMSON expression decreases the resistance of MCF-7dox cells (a doxorubicin-resistant breast cancer cell line) to doxorubicin and impedes the senescence escape of MCF-7 cells after doxorubicin treatment. Moreover, SAMMSON silencing in MCF-7dox cells induces a metabolic rewiring with a decrease in glycolysis and a concomitant increase in mitochondrial respiration related to a stimulation of mitochondrial protein translation. This study therefore sheds light on the possible modulation of the SAMMSON expression as an innovative therapeutic approach to targeting chemoresistant cancer cells specifically.

## 2. Materials and Methods

### 2.1. Cell Line and Cell Culture

The doxorubicin (DOX)-sensitive human breast cancer cell line MCF-7 was obtained from the American Type Culture Collection (ATCC^®^ HTB-22™, LGC Promochem, Molsheim, France). The doxorubicin-resistant human breast cancer cell line MCF-7dox was provided by Dr. K. Cowan (National Cancer Institute, Bethesda, MD, USA). This DOX-resistant cell line displays an IC50 approximately 500 times greater than that of MCF-7 cells. The cell lines were grown in Dulbecco’s modified Eagle’s medium (DMEM high glucose, Lonza, Basel, Switzerland) containing 10% fetal bovine serum (Gibco, Thermo Fisher Scientific, Waltham, MA, USA), 50 μg/mL of uridine, 100 μg/mL of sodium pyruvate and 2 mM glutamine (Thermo Fisher Scientific, Waltham, MA, USA), supplemented with 1 μM of DOX for the MCF-7dox line to maintain the multidrug-resistant phenotype. One week before experiments were performed, DOX was removed from the medium of MCF-7dox cells. All cell lines were tested for mycoplasma contamination using PCR detection (Venor^®^GeM, Minerva Biolabs, Berlin, Germany).

### 2.2. Small Interference RNA

Silencing of the lncRNA SAMMSON was performed on MCF-7dox cells using a predesigned siRNA directed against the target lncRNA (SAMMSON Silencer^®^ Select (n509333), Thermo Fisher Scientific, Waltham, MA, USA). This siRNA targets the exon 4 of SAMMSON and is predicted to silence five SAMMSON transcripts: ENST00000488861.7, ENST000000641336.1, ENST000000641286.1, ENST00000483525.2 and ENST000000671630.1 (Figure S1). The Scramble siRNA with no target was used as a control (Silencer^®^ Select Negative control # 1, Thermo Fisher Scientific, Waltham, MA, USA). Cells were reverse transfected using Lipofectamine as a transfecting agent (Lipofectamine^®^ RNAiMAX Reagent #13778075, Thermo Fisher Scientific, Waltham, MA, USA) and 30 pmol of SAMMSON or Scramble siRNA, according to the “Lipofectamine^®^ RNAiMAX Reagent Protocol 2013” from Invitrogen (Life Technologies, Thermo Fisher Scientific, Waltham, MA, USA). Forty-eight hours after transfection, cells were harvested for the experiments. The silencing of SAMMSON was verified for the different experiments by analyzing SAMMSON expression using quantitative PCR with two primer couples (SAMMSON1 and SAMMSON2, Table S1 and Figure S2). To further confirm the specificity of the siRNA used, the differential expression of one known target of SAMMSON, C1QBP (p32), and of its interaction network were checked in the transcriptional data of MCF-7dox siSAMMSON cells (Figure S3).

### 2.3. RNA Extraction, RT, qPCR

The total RNAs were extracted from 0.5 million cells with the RNAqueous™-Micro Kit (#AM1931 Invitrogen™, Thermo Fisher Scientific, Waltham, MA, USA) according to the manufacturer’s instructions. DNase I treatment was performed by adding directly to the RNA eluate 2 µL of DNase I buffer and 1 µL of DNase I. The mix was incubated for 30 min at 37 °C, and 2.3 µL of DNase Inactivation Reagent was added to the mixture. Reverse transcription of RNA into cDNA was performed on 2000 ng of total RNA using the SuperScript™ VILO™ cDNA Synthesis Kit ((#11754050 Invitrogen™, Thermo Fisher Scientific, Waltham, MA, USA). SAMMSON expression was assessed using quantitative PCR on a CFX Connect apparatus (Bio-Rad, Marnes-la-Coquette, France) and estimated using the relative quantitation method 2^-ΔΔCt^. GAPDH and 5S were used as reference genes. The sequences of the primers are shown in Supplementary Table S1.

### 2.4. Glucose and Lactate Measurements

Glucose and lactate measurements were carried out in the cell culture supernatants. The glucose concentration was measured using the Glucose Colorimetric Detection Kit (#EIAGLUC Invitrogen™, Thermo Fisher Scientific, Waltham, MA, USA) and lactate concentration was measured using the Lactate Assay Kit (#MAK064-1KT Sigma-Aldrich, Saint-Quentin-Fallavier, France) according to the manufacturer’s instructions.

### 2.5. Mitochondrial Respiration Rates

Mitochondrial oxygen consumption measurements were performed at 37 °C using a high-resolution oxygraph (O2k, Oroboros Instruments, Innsbruck, Austria) on 4 to 7 million intact cells, according to the method described in Desquiret-Dumas et al. [22]. In brief, cellular respiration was measured on intact cells in DMEM high-glucose medium supplemented with 1 mM glutamine (routine respiration). Phosphorylating respiration was then inhibited by adding 2 µg/mL of oligomycin (oligo respiration). Finally, respiratory chain maximal capacity was estimated by measuring respiration rates after sequential additions of the mitochondrial uncoupler FCCP (from 200 to 1400 nM) (FCCP respiration). Finally, non-mitochondrial respiration was checked by adding 2 µg/mL of antimycin A.

### 2.6. Mitochondrial ROS Production

ROS production was measured simultaneously with oxygen consumption at 37 °C in respiratory buffer RB using an O2k-Fluorometer (Oroboros Instruments, Innsbruck, Austria) equipped with a two-channel fluorescence optical setup to monitor oxygen level and fluorescence. H_2_O_2_ production was monitored using 10 μM of the H_2_O_2_-sensitive probe Amplex^®^ Red (Molecular Probes, Eugene, OR, USA), (excitation 525 nm/emission filter 580 nm) according to [22]. Then, 1 U/mL of horseradish peroxidase (HRP) and 5 U/mL of superoxide dismutase (SOD) were added to the chamber to convert superoxide into H_2_O_2_. Calibrations with stepwise additions of 0.1 μM H_2_O_2_ were carefully performed before, during and at the end of each experiment. ROS production and oxygen consumption were measured on 5 million digitonin-permeabilized cells using complex I (malate, pyruvate and glutamate) or complex II (succinate) substrates and ADP, according to the method described by Desquiret-Dumas et al. [22].

### 2.7. Mitochondrial Enzymatic Activities

The activities of the mitochondrial OXPHOS complexes were measured at 37 °C on a UVmc2 spectrophotometer (SAFAS, Monaco, Monaco) in a mitochondrial-enriched fraction from frozen cell pellets. The activities of NADH ubiquinone reductase (complex I), succinate ubiquinone reductase (complex II), ubiquinol cytochrome C reductase (complex III), cytochrome C oxidase (complex IV) and citrate synthase (CS) were measured according to the method described by Desquiret-Dumas et al. [22].

### 2.8. Western Blotting

Cellular proteins were solubilized in a Laemmli buffer, and 80 µg was resolved by SDS-PAGE in a 12% acrylamide gel. Proteins were then transferred to a nitrocellulose membrane in a semi-dry transfer apparatus (Bio-Rad, Marnes-la-Coquette, France). Mouse OXPHOS cocktail (Total OXPHOS human WB antibody cocktail (ab110411), Abcam), rabbit anti-TOMM20 (EPR15581-54 clone, ab186735, Abcam) and rabbit anti-αTubulin (EP1332Yclone, ab52866, Abcam) primary antibodies were used (1:1000 dilution). Membranes were incubated for two hours in the dark, with anti-mouse and anti-rabbit coupled, respectively, with Alexa Fluor 680 and Alexa Fluor 790 dyes (ab175775 and ab175781 Abcam, 1:20,000 dilution). Membranes were washed twice with TBS 1X–Tween 0.1% and once with TBS 1X. Fluorescence was detected at 700 and 800 nm with an Odyssey XF imaging system (LI-COR Biosciences, Bad Homburg, Germany). Band intensities were quantified with Image Studio software (LI-COR Biosciences, Bad Homburg, Germany).

### 2.9. RNA Sequencing

Gene expressions were measured on RNA extracts from siScb and siSAMMSON cell pellets using the Ion AmpliSeq™ Transcriptome Human Gene Expression Kit (A26325 Thermo Fisher Scientific, Waltham, MA, USA) according to the manufacturer’s instructions. In brief, cDNA was synthetized from 10 ng of total RNA using the SuperScript™ VILO™ cDNA Synthesis Kit (11754250 Thermo Fisher Scientific, Waltham, MA, USA). Up to 20,000 genes were amplified with multiplexed targeted AmpliSeq panels (Ion AmpliSeq™ Human Gene Expression Core Panel, Thermo Fisher Scientific, Waltham, MA, USA). After ligating the adaptors and barcodes, libraries were loaded on Ion 540™ chips using an Ion Chef™ apparatus (Thermo Fisher Scientific, Waltham, MA, USA). Libraries were sequenced on the Ion GeneStudio S5 next generation sequencer. Base calling, alignment and gene count steps were realized using the AmpliSeqRNA pipeline from the Ion Torrent v5.10 Suite (Thermo Fisher Scientific, Waltham, MA, USA).

### 2.10. Data Processing and Analysis

R v4.0.0 was used for data analysis. First, raw read counts were normalized using DESeq2 v1.28.0. After variance stabilizing transformation, a principal component analysis (PCA) showed differences in overall expression distributions according to the batch of the samples. To correct this, genes were removed if they showed low or no expressions (cutoff 20 counts) in all samples from a single batch. These data (7498 genes) were used for input, and ComBat-seq v3.36.0 was used to correct for the batch effect [23]. Finally, a PCA was used to confirm correction of the batch effect. Differential expression analysis was obtained using DESeq2, which normalizes each sample using size factors, employs a shrinkage estimator for gene variance and then uses a negative binomial model to test for differentially expressed genes [24]. The log2-fold change was estimated using the apeglm shrinkage estimator within DESeq2 [25]. The *p*-value was then computed for each gene and adjusted *p*-values were computed using a Benjamini–Hochberg correction with the false discovery rate at 0.05. Differentially expressed genes (DEG) were determined using a cutoff of *p*-value < 0.05. The list of mitochondrial genes (oxidative phosphorylation, mitochondrial translation, mitochondrial gene expression) was retrieved from the KEGG PATHWAY database (https://www.genome.jp/kegg/pathway.html, 27 July 2021) and PathCards (https://pathcards.genecards.org/, 27 July 2021). We used the R function heatmap2 to generate a heatmap of the mitochondrial DEG.

### 2.11. Cellular Growth Monitoring

The IncuCyte S3 live-cell imaging system (Essen BioScience) was used to monitor cell proliferation. Cells were seeded in 24-well plates at a density of 100,000 cells/well and transfected with the SAMMSON or Scramble siRNA for the MCF-7dox cells (MCF-7dox siScb, MCF-7dox siSAMMSON). Four hours later, they were treated with different concentrations of doxorubicin and placed in the IncuCyte S3 chamber. Real-time images of living cells were taken every 2 h for 2 days. To analyze cell proliferation, confluence was calculated by counting cells at the beginning and end of the experiment using ImageJ software.

### 2.12. Senescence Escape

MCF-7 cells were maintained in antibiotic-free RPMI-1640 medium (Lonza) supplemented with 10% FBS and maintained at 37 °C. Cells were cultured for 24 h in 6-well plates (50,000 cells) and treated for 96 h with doxorubicin (0.05 µM) in 3% FBS. Cells were then transfected with the SAMMSON or Scramble siRNA (50 nM) using DharmaFECT 4 according to the manufacturer’s instructions. For persistent cell generation, cells were washed with phosphate-buffered saline (PBS 1X) and restimulated with fresh 10% FBS for 10 days. Cells were then fixed with 2% formaldehyde for 15 min, washed with PBS 1X and stained with 0.4% crystal violet to count the number of clones that escaped senescence induced by the doxorubicin treatment.

### 2.13. Statistical Analysis

Data are represented as the mean ± SEM. The significance between groups was evaluated by the nonparametric Mann–Whitney test. All data were considered statistically significant at *p* < 0.05.

## 3. Results

### 3.1. SAMMSON Expression Increases the Resistance of MCF-7dox Cells to Doxorubicin and Promotes the Senescence Escape of MCF-7 Cells after Doxorubicin Treatment

**Figure 1:**
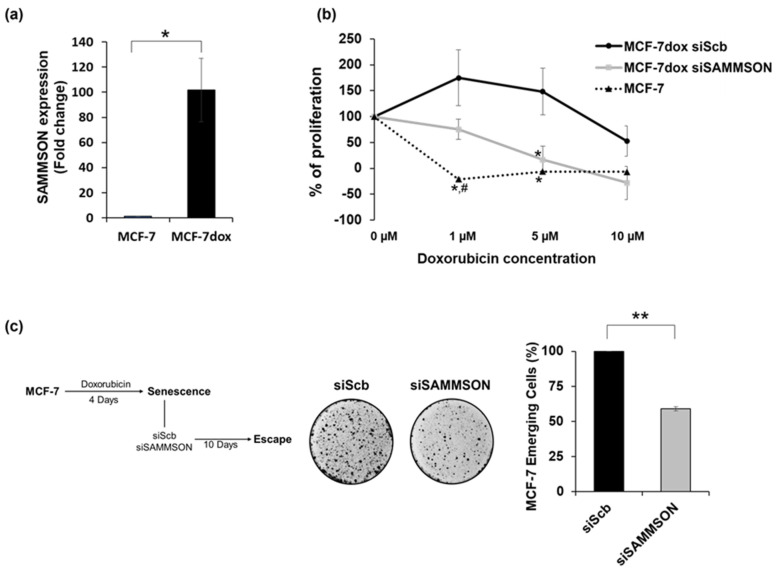
Expression and involvement of SAMMSON in the resistance of MCF-7dox cells to doxorubicin and in the senescence escape of MCF-7 cells after doxorubicin treatment. (**a**) RT-qPCR analysis of SAMMSON expression in doxorubicin-sensitive MCF-7 cells (MCF-7) and doxorubicin-resistant MCF-7dox cells (MCF-7dox). The data are represented by the mean ± SEM of *n* = 4 MCF-7 and MCF-7dox cells. (**b**) Doxorubicin sensitivity of MCF-7 cells (MCF-7) and MCF-7dox after SAMMSON silencing (siSAMMSON) compared to control cells MCF-7dox siScb (siScb). Cells were exposed to different doxorubicin concentrations (0, 1, 5 and 10 µM) and the number of cells was counted after 48 h and normalized to the number of cells counted at time 0 h. The data are represented by the mean ± SEM of *n* = 4 for MCF-7 cells and *n* = 5 for MCF-7dox siScb and siSAMMSON cells. (**c**) Senescence escape in MCF-7 cells treated for 96 h with doxorubicin and then transfected with SAMMSON (siSAMMSON) or Scramble siRNAs (siScb). Cell emergence was evaluated after 10 days. Left panel: representative images of the persistent cell stained with crystal violet; right panel: percentage of emerging cells. The *p*-values were calculated using the Mann–Whitney test; # indicates a significant difference compared to MCF-7dox siSAMMSON (# *p* < 0.05) and * indicates a significant difference compared to MCF-7dox siScb (* *p* < 0.05, ** *p* < 0.01).

We evaluated SAMMSON expression in doxorubicin-resistant MCF-7dox cells compared to doxorubicin-sensitive MCF-7 cells using quantitative PCR. SAMMSON overexpression was detected in the MCF-7dox cells (with a 101.8 ± 25.2-fold change, *p* = 0.021) in comparison to MCF-7 cells (Figure 1a). We first checked if SAMMSON inhibition per se modulated the proliferation rate of MCF-7dox cells by determining the doubling time of Scramble (MCF-7dox siScb) and SAMMSON (MCF-7dox siSAMMSON) siRNA transfected MCF-7dox cells. No variation of the doubling time was observed between the two conditions. This result was reinforced by a sulforhodamine B assay that disclosed no difference in protein amount between MCF-7dox siScb and MCF-7dox siSAMMSON cells (Figure S4). We then assessed the doxorubicin sensitivity of MCF-7 and MCF-7dox cells after Scramble (MCF-7dox siScb) or SAMMSON (MCF-7dox siSAMMSON) siRNA transfection. As expected, cell proliferation started to decrease at 1µM doxorubicin concentration for MCF-7 cells and beyond 5 µM for MCF-7dox cells (IC50 = 0.17 ± 0.03 µM for MCF-7 and 10.16 ± 0.86 µM for MCF-7dox cells, data not shown). Interestingly, at 5 µM doxorubicin concentration, we observed a stronger inhibition of cell proliferation in MCF-7dox siSAMMSON (16.3% ± 26.2; SAMMSON silencing results in 3 times significant decrease of IC50 value (3.13 ± 0.87 µM)) compared to MCF-7dox siScb cells (148.1% ± 45.2) (*p* = 0.03) (Figure 1b). We confirmed the potential link between SAMMSON expression and doxorubicin resistance by silencing SAMMSON in a senescence escape model of doxorubicin resistance in MCF-7 cells developed by Le Duff et al. [26,27,28]. In this model, by monitoring senescence markers the authors showed that after doxorubicin treatment, MCF-7 cells can enter senescence. They reported that a subpopulation of MCF-7 cells can adapt to chemotherapy-induced senescence and emerge as more transformed cells. For this purpose, MCF-7 cells were treated for 96h with doxorubicin, then transfected with the Scb or SAMMSON siRNA. Clone emergence was evaluated after 10 days and a 41% decrease in MCF-7 clones after SAMMSON silencing compared to siScb cells (*p* = 0.002) was observed (Figure 1c). Together, these data highlight the importance of the lncRNA SAMMSON in the chemoresistance of MCF-7 cells.

### 3.2. SAMMSON Modulates Complex I Activity and ROS Production in MCF-7dox Cells

**Figure 2:**
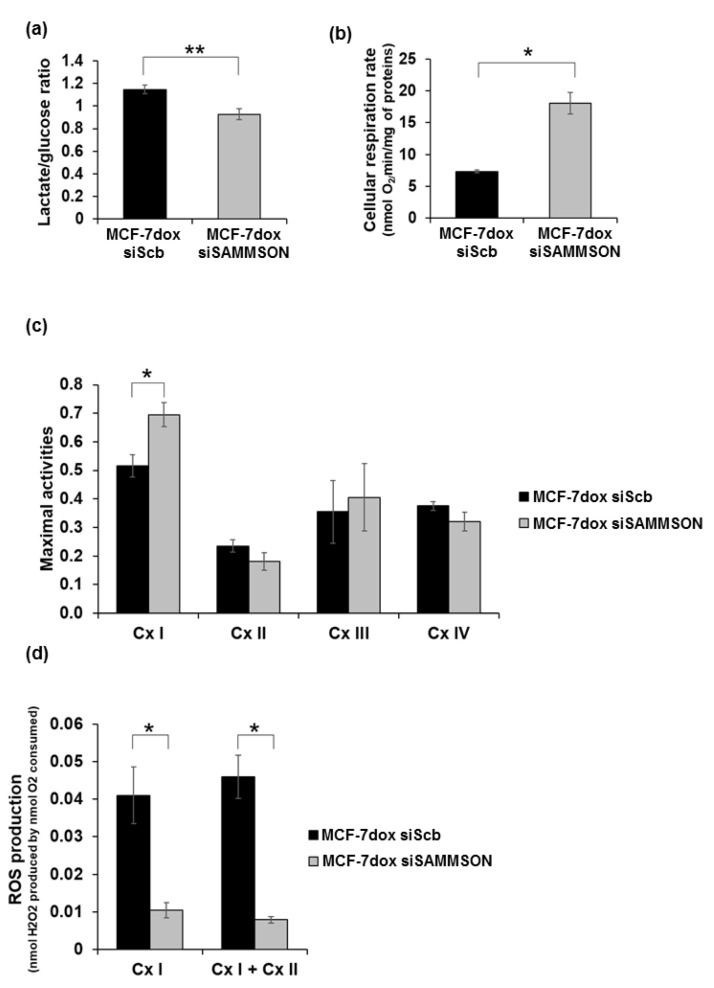
Evaluation of the metabolic orientation following SAMMSON silencing in MCF-7dox cells. (**a**) Estimation of glycolytic metabolism by calculating the ratio between lactate production and glucose consumption in MCF-7dox siScb and MCF-7dox siSAMMSON culture supernatants. The data are represented by the mean ± SEM of *n* = 6 for the MCF-7dox siScb and siSAMMSON cells. (**b**) Assessment of the oxidative metabolism on intact cells in glucose medium estimated by measuring the routine respiration rates in MCF-7dox siScb and MCF-7dox siSAMMSON cells. Raw data were normalized to the total amount of proteins. The data are represented by the mean ± SEM of *n* = 4 for the MCF-7 dox siScb and siSAMMSON cells. (**c**) Maximal activities of respiratory chain complexes I, II, III and IV were measured in duplicate on cell lysates from MCF-7dox siScb and siSAMMSON cells. Results were normalized to citrate synthase activity. Data are represented by the mean ± SEM of *n* = 7 for MCF-7dox siScb and siSAMMSON cell complex I activity and *n* = 5 for MCF-7dox siScb and siSAMMSON cell complex II, III and IV activities. (**d**) Measurement of ROS production normalized to the corresponding oxygen consumption using high-resolution respirometry on permeabilized cells. Dedicated Cx I and Cx I+Cx II linked ROS production was sequentially analyzed using substrates of Cx I and Cx I + Cx II. The data are represented by the mean ± SEM of *n* = 4 for MCF-7dox siScb and MCF-7dox siSAMMSON cells. The *p*-values were calculated using the Mann–Whitney test, with * *p* < 0.05 and ** *p* < 0.01.

We then assessed the metabolic orientation of MCF-7dox following SAMMSON silencing. First, we evaluated the glycolytic metabolism by measuring the ratio between the glucose consumption and lactate production in a culture medium of MCF-7dox siScb and MCF-7dox siSAMMSON cells. We observed a 19% decrease in the lactate/glucose ratio in MCF-7dox siSAMMSON cells (*p* = 0.008), which underlies a decreased glycolytic metabolism following SAMMSON silencing (Figure 2a). We next assessed the oxidative metabolism by monitoring cellular endogenous respiration using high-resolution oxygraphy on intact cells. Results showed a 145% increase in the routine respiration rate in MCF-7dox siSAMMSON cells (18.09 ± 1.68 nmol O^2^/min/mg of protein) compared to MCF-7dox siScb cells (7.36 ± 0.25 nmol O^2^/min/mg of protein) (*p* = 0.029) evidencing a higher oxidative metabolism following SAMMSON silencing in MCF-7dox cells (Figure 2b).

We next sought to further investigate the increase in oxidative metabolism following SAMMSON silencing by measuring the maximal enzymatic activities of mitochondrial complexes on cell extracts from MCF-7dox siScb and siSAMMSON cells. Notably, we observed a 1.35-fold increase in the maximal specific activity of complex I (Cx I) in MCF-7dox siSAMMSON cells (0.70 ± 0.04) compared to MCF-7dox siScb cells (0.52 ± 0.04) (*p* = 0.029). In contrast, we did not observe any variation in the maximal activities of Cx II, Cx III and Cx IV following SAMMSON silencing (Figure 2c).

Complex I is the largest component of the mitochondrial respiratory chain and is one of the main sources of ROS production. To determine whether the stimulation of Cx I activity following SAMMSON silencing influences the production of ROS, we measured it in parallel with the maximal respiration rate on permeabilized cells in the presence of Cx I and Cx I + Cx II substrates. SAMMSON silencing induced a significant 4-fold decrease in maximal Cx I ROS production in MCF-7dox siSAMMSON cells (0.010 ± 0.002 nmol H_2_O_2_ produced per nmol O_2_ consumed) compared to MCF-7dox siScb cells (0.041 ± 0.007 nmol H_2_O_2_ produced per nmol O_2_ consumed) (*p* = 0.028) (Figure 2d). A similar result was observed for Cx I + Cx II in MCF-7dox siSAMMSON cells (0.008 ± 0.001 nmol H_2_O_2_ produced per nmol O_2_ consumed) compared to MCF-7dox siScb cells (0.046 ± 0.006 nmol H_2_O_2_ produced per nmol O_2_ consumed) (*p* = 0.028). Therefore, these data emphasize a major role of SAMMSON in regulating Cx I activity and ROS production.

### 3.3. SAMMSON Participates in Mitochondrial Replication, Transcription and Translation in MCF-7dox Cells

**Figure 3:**
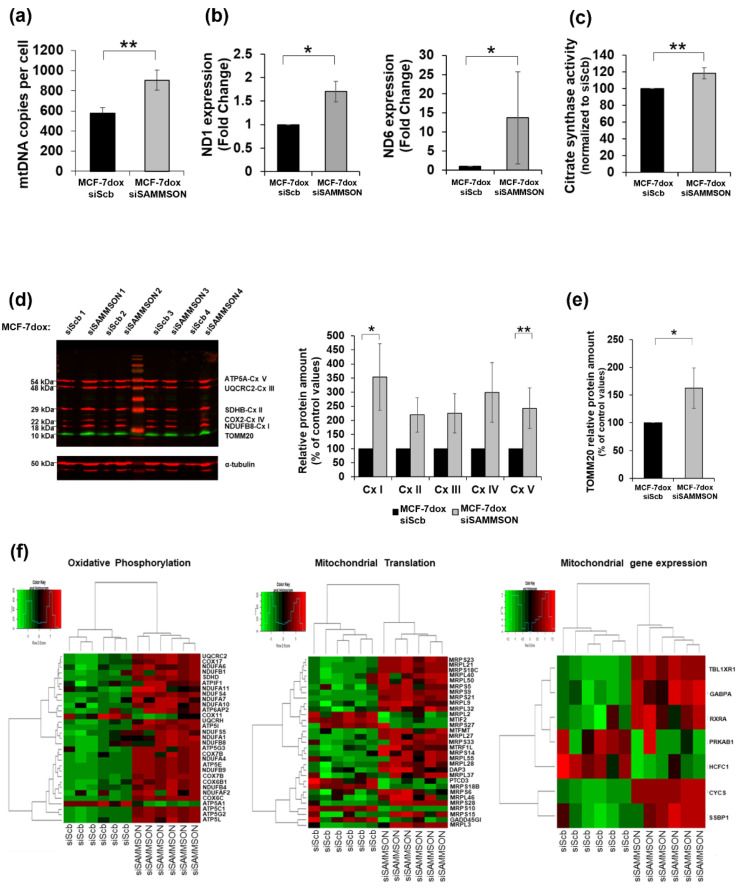
Impact of SAMMSON silencing in mitochondrial replication, transcription and translation in MCF-7dox cells. (**a**) Quantification of the mitochondrial DNA (mtDNA) in the MCF-7dox siScb and siSAMMSON cells. The ratio of the mtDNA/nuclear DNA was analyzed using qPCR. The data are represented by the mean ± SEM mtDNA copies per cell of *n* = 5 MCF-7dox siScb and siSAMMSON cells. (**b**) Quantification of the mitochondrial RNA ND1 and ND6 in the MCF-7dox siScb and siSAMMSON cells via RT-qPCR. Data are represented by the mean ± SEM of *n* = 4 MCF-7dox siScb and siSAMMSON cells. (**c**) Citrate synthase activity was measured in duplicate on crude cell lysates and normalized to the total amount of proteins. The data are represented by the mean ± SEM of *n* = 8 MCF-7dox siScb and siSAMMSON cells. (**d**) Western blot of OXPHOS mitochondrial complexes performed on MCF-7dox siScb and siSAMMSON cell lysates. An antibody cocktail was used to examine the expression of five mitochondrial proteins. Left panel: representative blot; right panel: quantification of respiratory chain complexes (Cx I, Cx II, Cx III, Cx IV and Cx V) normalized to α-tubulin protein quantity. Data are represented by the mean ± SEM of n = 6 for MCF-7dox siScb and siSAMMSON cells. (**e**) Quantification of the mitochondrial marker TOMM20 normalized to α-tubulin. (**f**) Heatmap of differentially expressed genes in mitochondrial pathways in MCF-7dox siScb and siSAMMSON treated cells. For panels (**a**–**e**), *p*-values were calculated using the Mann–Whitney test, with * *p* < 0.05 and ** *p* < 0.01.

To obtain greater detail regarding the mechanism linking SAMMSON inhibition and the stimulation of the oxidative metabolism in MCF-7dox cells, we estimated the mitochondrial mass using various approaches. We first evaluated the mitochondrial DNA (mtDNA) copy number per cell using quantitative PCR. The silencing of SAMMSON resulted in a 1.58-fold increase in the amount of mtDNA per cell in MCF-7dox cells (906 ± 101 mtDNA copies per cell in MCF-7dox siSAMMSON cells compared to 575 ± 55 mtDNA copies per cell in MCF-7dox siScb cells, *p* = 0.008) (Figure 3a). We then assessed mitochondrial Cx I subunit expressions using quantitative PCR and found that SAMMSON silencing resulted in a significant increase of ND1 and ND6 RNA expression in MCF-7dox siSAMMSON cells (1.70 ± 0.22- and 13.67 ± 12.10-fold changes, respectively) compared to MCF-7dox siScb cells (*p* = 0.021) (Figure 3b). Following this, we measured the activity of citrate synthase (CS), which reflects the mitochondrial mass. Notably, CS activity showed a 1.18-fold increase in MCF-7dox siSAMMSON cells (*p* = 0.008) (Figure 3c). We analyzed the quantity of mitochondrial respiratory chain complexes and the mitochondrial outer membrane protein TOMM20 protein amounts using the Western blot technique. We observed a 3.53-fold increase in the quantity of the NDUFB8 Cx I subunit (*p* = 0.049) and a 2.43-fold increase in the quantity of the ATP5B Cx V subunit (*p* = 0.003) in MCF-7dox siSAMMSON cells (Figure 3d). Similarly, in these cells, although not statistically significant, we noticed a slight increase in the abundance of the Cx II, Cx III and Cx IV subunits. In addition, the quantity of TOMM20 was 1.62-fold higher in MCF-7dox siSAMMSON cells compared to MCF-7dox siScb cells (*p* = 0.049) (Figure 3e). Transcriptome analyses of MCF-7dox siSAMMSON cells compared to MCF-7dox siScb cells also revealed that oxidative phosphorylation (OXPHOS) and mitochondrial translation were significantly impacted by SAMMSON inhibition (Figure 3f). Strikingly, 13 nuclear-encoded Cx I subunit genes were overexpressed in MCF-7dox siSAMMSON cells (NDUFA6, NDUFB1, NDUFA11, NDUFS4, NDUFA7, NDUFA10, NDUFS5, NDUFA1, NDUFB8, NDUFA4, NDUFB9, NDUFB4 and NDUFAF2). Regarding the mitochondrial translation pathway, a large number of mitochondrial ribosome proteins involved in mitochondrial protein synthesis were also overexpressed in MCF-7dox siSAMMSON cells (MRPS23, MRPL21, MRPS18C, MRPL40, MRPL50, MRPS5, MRPS9, MRPL32, MRPL2, MRPS27, MRPL27, MRPS33, MRPS14, MRPL55, MRPL28, MRPL37, MRPS18B, MRPS6, MRPL46, MRPS28, MRPS10, MRPS15 and MRPL3). Altogether, these results revealed an increased mitochondrial replication, supported by an increased transcription of several subunits of the mitochondrial respiratory chain complexes and of protein translation pathways, following SAMMSON silencing.

## 4. Discussion

In the present study, we analyzed the role of SAMMSON in metabolism and chemoresistance in the doxorubicin-resistant breast cancer MCF-7dox cell line. Interestingly, we found that SAMMSON is overexpressed in these cells in comparison to doxorubicin-sensitive MCF-7 cells. We demonstrated that SAMMSON silencing decreases the resistance of MCF-7dox cells to doxorubicin and reduces the senescence escape of MCF-7 cells after doxorubicin treatment. Our results also highlighted that SAMMSON silencing counteracts the Warburg effect in MCF-7dox cells, inducing a decrease in glycolysis and conversely increasing mitochondrial respiration. In addition, our results suggest that SAMMSON silencing impacts mitochondrial replication, transcription and translation while also targeting Cx I by increasing its activity.

SAMMSON expression is upregulated and associated with a poor prognosis in melanoma, oral squamous cell carcinoma and liver cancers [13,14,15]. The ability to adapt the cellular metabolism to stress conditions, a process called metabolic plasticity, is an important feature of cancer cells and a crucial property of chemoresistant cells [29,30]. We hypothesize that the effect of SAMMSON that we disclosed on MCF-7dox chemoresistance is linked to its effect on cellular metabolism. In this context, inhibiting SAMMSON in resistant cells appears to be a promising way to induce metabolic rewiring of the oxidative metabolism, a condition that should ultimately increase cellular sensitivity to chemotherapy. Indeed, the glycolytic metabolism is a determinant of the aggressiveness and chemoresistance of cells. Lactic acid produced by glycolysis promotes cytosolic acidification and hypoxia, conferring a phenotype resistant to cancer cells [31]. In breast cancer cells, an enhanced rate of glycolysis was evidenced in drug-resistant cells compared to sensitive cells [32]. Furthermore, targeting glycolysis by targeting glucose transporters or enzymes of the glycolytic pathway can reverse chemoresistance [33]. Mitochondrial impairment was recently described in MCF-7dox cells with a predominant glycolytic metabolism, with a strong impairment of Cx I structure and function [34]. Our study further demonstrates that silencing SAMMSON in this model not only enables us to slow glycolysis down, but it also triggers a stimulation of the oxidative metabolism by increasing mitochondrial translation pathways. A relationship between SAMMSON and the mitochondrial metabolism has already been described in melanoma cells [13,18]. In these studies, SAMMSON silencing by antisense oligonucleotides resulted instead in a decrease in mitochondrial protein synthesis [18]. Both the particular feature of the metabolism in chemoresistant cells and the cellular type itself might explain the discrepancy between these results and those that we have presented here. Regardless of the pathways involved, the previous studies on melanoma cells [13,18] and our own data all demonstrate that SAMMSON is an important regulator of cellular metabolic orientation and plays a role in tumor outcome by providing an advantage to the cancer cell and favoring its survival in both tumor progression [18,35] and chemoresistance.

**Figure 4:**
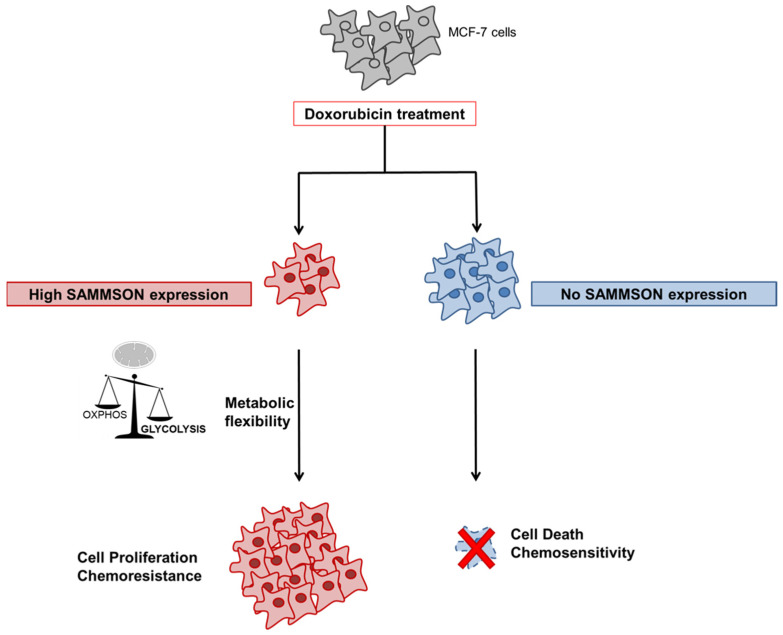
Schematic representation of the impact of SAMMSON on cellular metabolism and chemoresistance in MCF-7 cells. After doxorubicin treatment, in a pool of cells, high SAMMSON expression favors the Warburg effect with increased glycolysis and decreased mitochondrial respiration, resulting in increased resistance of these cells to doxorubicin.

Research has recently provided evidence of a high ROS production in MCF-7dox cells [34]. In the present study, we showed that the silencing of SAMMSON decreased this ROS production. Anthracyclines such as doxorubicin are a widely used class of chemotherapy drugs that inhibit the topoisomerase II enzyme, resulting in damage to DNA and cell death [36]. Doxorubicin can also target the mitochondrial topoisomerase Top1mt that alters mtDNA integrity and increases ROS production [37]. This process leads to mitochondrial dysfunctions, which in turn increase ROS production and glycolysis, consequently leading to chemoresistance [38]. In normal conditions, ROS overproduction causes cell damage, leading to the activation of apoptosis [39]. However, cancer cells have developed adaptations to overwhelm the oxidative stress through a high expression of antioxidant compounds [40]. This high ROS level stimulates cancer cell progression both by activating cell survival pathways (Akt, mTOR and NF-κB) and by stimulating angiogenesis related to hypoxia (HIF-1α) [40]. Moreover, the modulation of ROS levels by cancer cells is an important step in the acquisition of chemoresistance. Some twenty lncRNAs have been linked to chemoresistance in breast cancer cells [41,42,43,44,45,46,47,48,49,50,51,52,53], but to the best of our knowledge none has been involved in the modulation of ROS production. In our model of breast cancer cells resistant to doxorubicin, the expression of SAMMSON could be part of the cellular stress response favoring ROS production and metabolic switching (Figure 4).

In this context, the inhibition of SAMMSON expression would, via the increase of the Cx I activity and ROS inhibition that we showed in this study, restore an oxidative metabolism and counterbalance the glycolytic pathway, therefore contributing to decreasing chemoresistance. These results should prompt the study of the mechanism of SAMMSON on the Cx I activity in this cell model. Furthermore, SAMMSON shows itself to be an attractive therapeutic target for monitoring the metabolic reorientation and decreasing chemoresistance.

## 5. Conclusions

The results presented in this paper highlight the role of the intergenic long non-coding RNA SAMMSON in conferring to MCF7 breast cancer cells the metabolic plasticity indispensable to resistance to chemotherapeutic agents. This long non-coding RNA therefore appears as an important link between metabolic orientation and survival in cancer cells. Modulation of SAMMSON expression in breast cancer cells could therefore represent an interesting therapeutic lever to improve chemotherapeutic treatment efficiency while avoiding off-target effects on non-cancerous adjacent cells that do not express this long non-coding RNA.

## Data Availability

The data presented in this study are available on request from the corresponding author. Count data for RNA-seq are deposited in the Gene Expression Omnibus (GEO) repository (GSE188404).

## Supplementary Materials

The following are available online at https://www.mdpi.com/article/10.3390/biology10111156/s1, Table S1: Primer and siRNA sequences, Figure S1: Location of siRNA sequence of SAMMSON isoforms listed in ENSEMBL, Figure S2: Detection of SAMMSON expression inhibition by siRNA, Figure S3: Expression of SAMMSON targets after SAMMSON silencing by siRNA, Figure S4: Impact of SAMMSON silencing in MCF7-dox cells proliferation in absence of doxorubicin.
