# Supplementary material for: The Long Non-Coding RNA SAMMSON Is a Regulator of Chemosensitivity and Metabolic Orientation in MCF-7 Doxorubicin-Resistant Breast Cancer Cells
Source: Biology (Basel). 2021 Nov 9;10(11):1156. doi: 10.3390/biology10111156 (PMC8615054; doi:10.3390/biology10111156)
Supplement: Supplementary file 1 [file biology-10-01156-s001.zip › biology-1414663-supplementary.pdf]

Supplemental Material : siRNA characterisation

Table 1: Primer and siRNA sequences

Target		Sequence (5' to 3')	Reference
SAMMSON 1	Forward	TTCCTCAACTATAGCAACTCAA	(Shao et al., 2020; Zheng et al., 2020)
	Reverse	TAGACTACGGGCTCATTACTT	
SAMMSON 2	Forward	CCTCTAGATGTGTAAGGGTAGT	(Vendramin et al., 2018)
	Reverse	TTGAGTTGCATAGTTGAGGAA	
GAPDH	Forward	AGCCACATCGCTCAGACAC	(Peng et al., 2014)
	Reverse	GCCCAATACGACCAAATCC	
5S	Forward	GGCCATACCACCCTGAACG	(Zonneville et al., 2020)
	Reverse	AGCCTACAGCACCCGGTATT	
SAMMSON siRNA	Sense	CAGUCAAAACAAAACCAUUtt	ThermoFisher n509333
	Antisense	AAUGGUUUUGUUUUGACUGca	

Figure S1: Location of siRNA sequence of SAMMSON isoforms listed in ENSEMBL

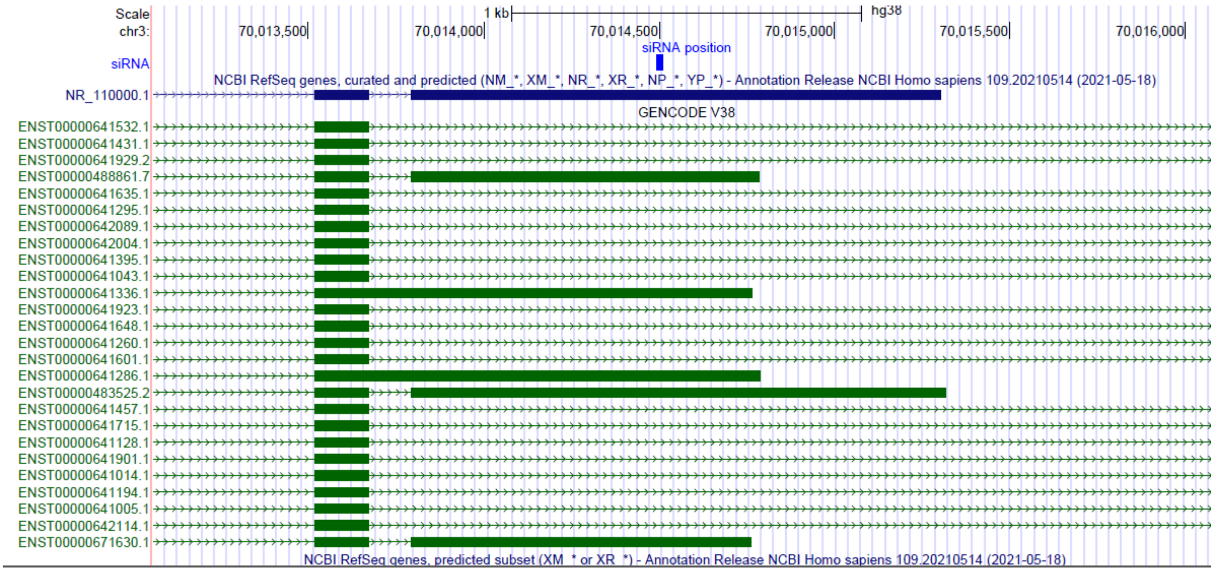

SAMMSON ENSEMBL tracks were uploaded in UCSC Genome browser (https://genome.ucsc.edu/) to visualize the different exons of each transcript and the siRNA location was positioned on these tracks. The siRNA used in this study targets 5 SAMMSON transcripts: ENST00000488861.7, ENST000000641336.1, ENST000000641286.1, ENST00000483525.2 and ENST000000671630.1.

Figure S2: Detection of SAMMSON expression inhibition by siRNA

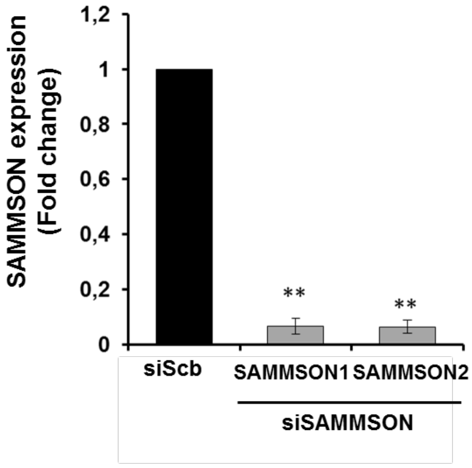

SAMMSON expression was detected by quantitative PCR in MCF-7dox siScb and siSAMMSON cells. Two primers couples were used to quantify SAMMSON expression (SAMMSON 1 and SAMMSON2, the sequences are detailed in Supplemental Material Table 1, n=4, **: *p<0.01* compared with MCF-7dox siScb cells).

Figure S3: Expression of SAMMSON targets after SAMMSON silencing by siRNA

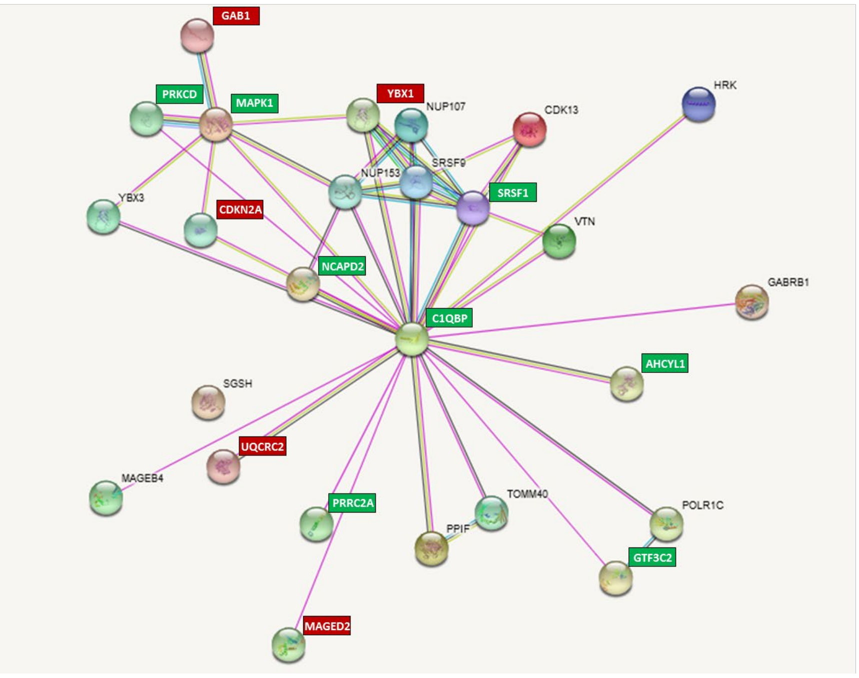

	FC	padj
C1QBP	-5.30	0.009
GAB1	1.76	0.010
PRKCD	-2.98	0.022
MAPK1	-1.49	5.43E-05
CDKN2A	0.72	4.17E-27
NCAPD2	-2.50	7.70E-11
YBX1	3.06	0.022
SRSF1	-4.79	0.001
AHCYL1	-1.85	2.37E-15
GTF3C2	-1.72	0.001
MAGED2	1.34	6.47E-08
PRRC2A	-4.28	0.038
UQCRC2	1.78	4.76E-09

Regulation of the expression of C1QBP (SAMMSON target) and of its interaction network after SAMMSON silencing in MCF-7dox cells determined by transcriptional analysis. Overexpressed genes are in a red square and underexpressed genes are in a green square. (FC: fold change; padj : p value adjusted).

Figure S4: Impact of SAMMSON silencing in MCF-7dox cells proliferation in absence of doxorubicin

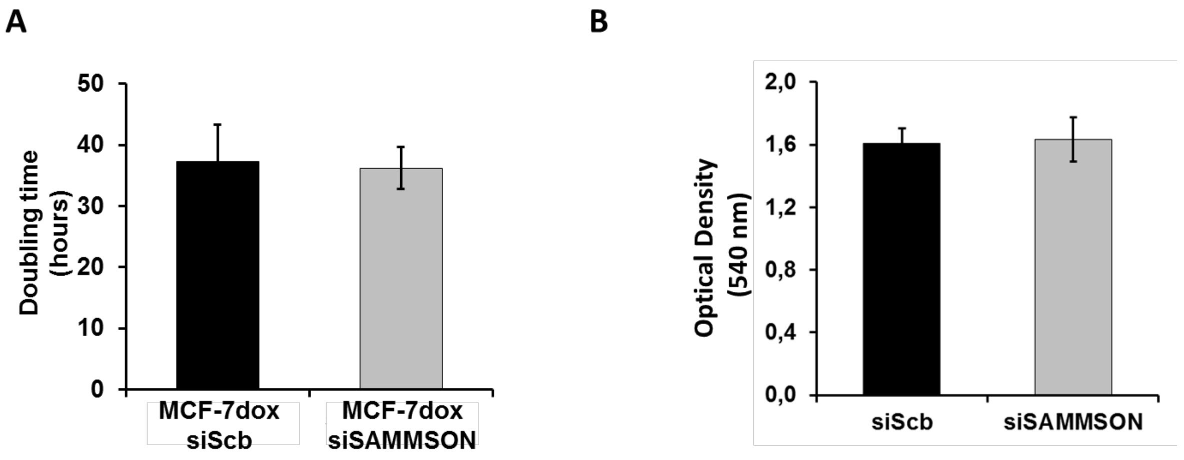

A. Doubling time of MCF-7dox siScb and siSAMMSON cells were calculated from proliferation measurement data (n=5). **B.** Cell protein content estimation by sulforhodamine B (SRB) assay in MCF-7dox siScb and siSAMMSON cells.

References:

Peng, C., Zhang, Z., Wu, J., Lv, Z., Tang, J., Xie, H., Zhou, L., and Zheng, S. (2014). A critical role for ZDHHC2 in metastasis and recurrence in human hepatocellular carcinoma. Biomed Res Int 2014, 832712.

Shao, L., Sun, W., Wang, Z., Dong, W., and Qin, Y. (2020). Long noncoding RNA SAMMSON promotes papillary thyroid carcinoma progression through p300/Sp1 axis and serves as a novel diagnostic and prognostic biomarker. IUBMB Life 72, 237-246.

Vendramin, R., Verheyden, Y., Ishikawa, H., Goedert, L., Nicolas, E., Saraf, K., Armaos, A., Delli Ponti, R., Izumikawa, K., Mestdagh, P., et al. (2018). SAMMSON fosters cancer cell fitness by concertedly enhancing mitochondrial and cytosolic translation. Nat Struct Mol Biol 25, 1035-1046.

Zheng, X., Tian, X., Zhang, Q., Shi, P., and Li, S. (2020). Long non-coding RNA SAMMSON as a novel potential diagnostic and prognostic biomarker for oral squamous cell carcinoma. J Dent Sci 15, 329-335.

Zonneville, J., Wong, V., Limoge, M., Nikiforov, M., and Bakin, A.V. (2020). TAK1 signaling regulates p53 through a mechanism involving ribosomal stress. Sci Rep 10, 2517.
